# Prevalence of Human Papillomavirus (HPV) in the Oral Cavity of a Healthy Population in South-Eastern Poland

**DOI:** 10.3390/ijerph19127213

**Published:** 2022-06-12

**Authors:** Marcin Koleśnik, Ewa Stępień, Małgorzata Polz-Dacewicz

**Affiliations:** Department of Virology with SARS Laboratory, Medical University of Lublin, 20-093 Lublin, Poland; ewa.stepien@umlub.pl (E.S.); malgorzatapolzdacewicz@umlub.pl (M.P.-D.)

**Keywords:** HPV, prevalence, healthy population, oropharyngeal cancer, oral, papillomavirus

## Abstract

Human papillomavirus (HPV) is associated with both benign lesions and with neoplastic diseases, such as oropharyngeal cancer. Due to the increasing occurrence of these neoplasms on a global scale, it seems important to estimate the risk factors in the population. So far, data on the presence of oral HPV in the European population are scarce. Oral HPV was detected in 53 out of 461 healthy subjects (11.5%) in south-eastern Poland. Among the most common oncogenic types, HPV 16 was reported in four subjects (0.87%) and HPV 18 in three subjects (0.65%). Another high-risk type, HPV 45, was detected in nine subjects (1.95%). Low risk types (HPV 6, 11, 54) were detected in 25 cases, which constituted 5.4% of all tested samples. In adults and children, statistically more HPV positive cases have been reported in males than in females. A positive relationship was demonstrated between age and the amount of positive oral HPV. The presence of HPV was statistically more frequent in the population living in urban rather than in rural areas in all studied age groups (*p* < 0.05).

## 1. Introduction

Human papillomaviruses (HPV) constitute a genotypically diverse group of small nonenveloped, double-stranded DNA viruses that are mainly transmitted through sexual contact. HPV infects the epithelial cells of the skin and mucosa. Most HPV infections are asymptomatic and are cleared within 12–24 months after infection. However, some of these infections may persist and become life-threatening [1,2,3,4]. The HPV genotypes are grouped into different genera: Alpha-, Beta-, Nu-/Mu-, and Gamma-papillomavirus according to tissue tropism and genome structure [3]. Moreover, HPV can be classified according to the potential for oncogenesis [2,5]. More than 200 HPV genotypes have been identified and classified into those with low (low-risk) and high (high-risk) oncogenic potential. The International Agency for Research on Cancer has classified twelve HPV types (16, 18, 31, 33, 35, 39, 45, 51, 52, 56, 58, and 59) as carcinogenic to humans [1]. These types are associated with head and neck, cervical and anogenital tumors. Among the high-risk types, HPV 16 and HPV 18 are the most common genotypes found in all HPV-related cancers [1,2,6]. HPV can induce carcinogenic transformation of infected mucosal epithelium by E6 and E7 oncoproteins, which inhibit p53 and pRB tumor suppressors [3]. Cancer usually develops over the years or even decades after a person becomes infected with HPV. By contrast, low-risk HPV including HPV 6 or HPV 11 primarily causes skin and mucosal changes, such as warts, premalignant lesions, or laryngeal papillomatosis. Environmental, host, and viral factors can influence any stage of HPV infection. For example, the incidence of HPV infection is higher in immunocompromised individuals than in the general population. People with a weakened immune system have an increased risk of developing HPV-related tumors, characterized by aggressive and rapid growth. In addition, the low rate of viral replication in basal cells prevents the immune system from acting against the virus [1,3,5,7,8].

The presence of oral HPV prevalence can cause a wide variety of lesions, from benign to malignant. One of the most common cancers in the head and neck region is oropharyngeal squamous cell carcinoma. HPV infects the mouth and throat, causing cancer of the oropharynx that covers the back of the throat, including the base of the tongue and tonsils [9]. It is characterized by significant morbidity and mortality despite the use of invasive treatment. Oropharyngeal cancer incidence rates are increasing worldwide and are associated with oral HPV infection, especially HPV 16. Additionally, there are large geographic variations in HPV-related oropharyngeal cancer. The most important predictors of oral high risk-HPV infection are sexual habits and smoking. In addition, the use of hormones or coinfections with HIV may contribute to the progression of HPV infection to cancer. Nowadays, knowledge of the prevalence of oral HPV infections is also particularly significant with the ongoing debate about HPV vaccination for men [1,10]. The presence of oral HPV in the healthy population has been investigated in many studies on different continents; however data in Europe is limited. Better understanding of its epidemiology and infection-related characteristics is crucial for effective and targeted prevention efforts [11].

The aim of this study is to assess the prevalence of HPV in the oral cavity of a healthy population in south-eastern Poland. Information on the presence of specific genotypes in a healthy population and the examination of the correlation between viral infection and socio-demographic features is essential for future screening and early diagnosis of the oral lesions, HPV-related cancers, and other comorbidities.

## 2. Materials and Methods

### 2.1. Study Group

The present study involved a total of 461 healthy people, including 118 children aged 5–10 years, 97 adolescents aged 15–19 years, and 246 adults over 20 years of age. Oral swabs were collected from all individuals. Brush swabs were collected by a dentist during a preventive dental examination. Admission criteria included no head and neck neoplasm or precancerous lesions, no lesions associated with HPV infection, no previous diagnosis of anal or genital warts or tumors, no severe systemic condition or HIV, and good general health. People were divided according to the place of residence into rural or urban areas and classified into age groups. Additionally, the respondents were divided according to gender.

### 2.2. Oral Brush Swab Collection

A cytobrush was rubbed with medium pressure over the premolar and molar region of the cheeks 5 times, turned, and rubbed another 5 times. This procedure was performed both on the right and left cheeks. The cytobrush was then placed in an Eppendorf tube and frozen at −80 °C until the analysis.

### 2.3. DNA Extraction/HPV Detection and Genotyping

The viral genetic material was isolated using the QIAamp DNA Mini Kit (Qiagen, Hilden, Germany) according to the manufacturer’s instructions. After isolation, the purity and yield of the obtained eluate were assessed using an Epoch spectrophotometer (Biotek Instruments, Winooski, VT, USA). The measurement was performed on the Take 3 plate (Biotek Instruments, Winooski, VT, USA) enabling measurement in microvolumes (2 μL). Microplate Reader Software Gen 5.2.0 (Biotek Instruments, Winooski, VT, USA) was used to analyze the results. The isolates were stored at −20 °C until the test was performed. In order to check the quality of the obtained DNA (presence of polymerase chain reaction inhibitors), a test for β-globin was performed.

HPV detection and genotyping were performed under the INNO-LiPA HPV Genotyping Extraassay (Innogenetics, Gent, Belgium) according to the manufacturer’s protocol. The kit is based on the amplification of a 65 bp fragment from the L1 region of the HPV genome with a SPF10 primer set. PCR products are subsequently typed with the reverse hybridization assay. As a quality control, primers amplifying a fragment of the human HLA-DPB1 gene are added to the HPV amplification kit to check the quality of the sample and the efficiency of the extraction. The quality control also used positive and negative control and conjugate control line. Assay includes all currently known high risk HPV genotypes and probable high risk HPV genotypes (16, 18, 26, 31, 33, 35, 39, 45, 51, 52, 53, 56, 58, 59, 66, 68, 73, 82). In addition, this kit allows the detection of low-risk HPV genotypes (6, 11, 40, 43, 44, 54, 70) and some additional genotypes (69, 71, 74). After DNA extraction, the samples were amplified by PCR (40 cycles) using Inno-LiPA HPV Genotyping Extra Amp according to the manufacturer’s instructions.PCR amplification was performed in a reaction mixture including excess deoxynucleoside 5’-triphosphates (dNTPs) containing deoxyuridine triphosphate, biotinylated primers, thermostable DNA polymerase, and uracil-N-glycosylase (UNG). After 40 cycles of PCR reaction, the multi-amplified biotinylated target sequence was acquired. Thereafter, hybridization of the amplified product was performed on the strip, followed by thorough washing. Conjugate and substrate were then added, causing color development. The strips were read visually using a manufacturer’s reference guide when they were completely dry.

### 2.4. Statistical Analysis

The socio-demographic characteristics of the patients and the difference in the frequency of HPV between different groups of people were determined using the Pearson chi-square test. The statistical significance was defined as *p* < 0.05.

### 2.5. Ethics

The research was approved by the Medical University of Lublin Ethics Committee and is in accordance with the GCP (Good clinical practice) regulations (no. KE-0254/133/2013, 23 May 2013). Written consent was obtained from the respondents and from parents in the case of children and youth. 

## 3. Results

**Figure 1:**
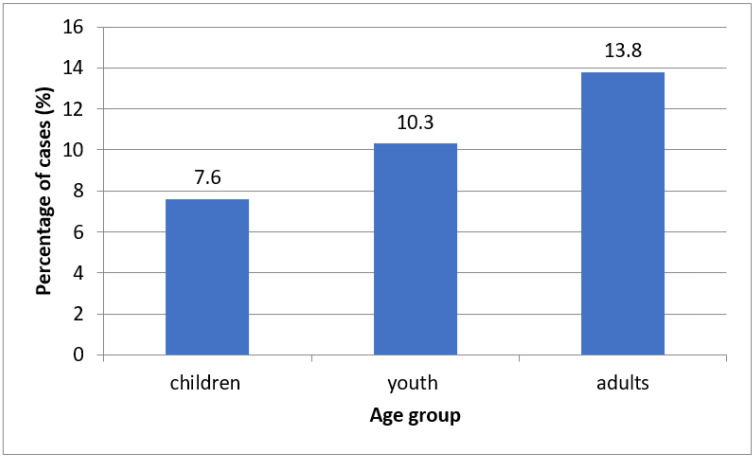
The frequency of positive HPV cases in the oral cavity of the studied groups. Children (age 5–10); youth (age 11–19); adults (age 20–39; 40–59; 60+).

**Figure 2:**
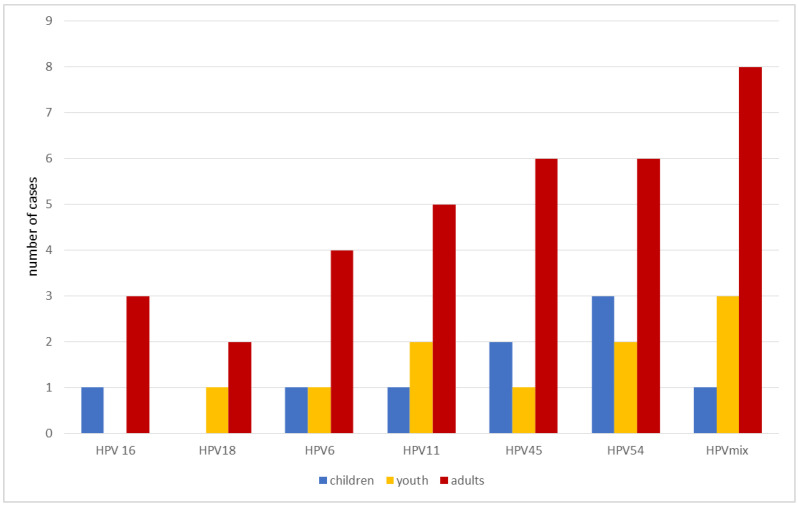
Presence of different HPV genotypes by age groups. HPV mix (HPV 45, HPV 54, HPV 16, HPV 18). An HPV mix can be defined as several genotypes simultaneously. Children (age 5–10); youth (age 11–19); adults (age 20–39; 40–59; 60+).

The presence of HPV was statistically more common in the population living in the urban area in all studied age groups. In adults and children, statistically more HPV positive cases have been reported in men than in women. Additionally, in the case of youth, more HPV positive cases in men than in women were not statistically significant (Table 1). Overall presence of HPV positive cases was 11.5% (53/461). The highest percentage of HPV DNA was detected in the oral cavity of adults (13.8%) and the lowest in children (7.6%) (Figure 1, Table 1). In the adult group, the percentage of HPV positive among the total cases of their own subgroup was 7.3% for the 20–39 age group, 10.3% for the 40–59 age group, and 23.3% for the 60+ age group, respectively.In contrast, 10.3% of HPV cases were reported in the youth group. In adults, the HPV mix constituted 3.2% (8/246) of all subjects in this group, which was the most frequently detected genotype. The HPV 18 genotype in the same group was detected in 0.8% (2/246), being the least frequently detectable. Taking into account only positive results, the cases in this group have an HPV mix percentage of 23.5% (8/34). For HPV 45 and HPV 54, the same number of cases was reported and their percentage of positive adults was 17.7% (6/34). The percentage of remaining genotypes in this group of positive cases was 14.7% for HPV 11, 11.8% for HPV 6, 5.9% for HPV 18, and 8.82% for HPV 16, respectively. Moreover, in this group, there was a positive correlation between age and the number of positive HPV cases in the oral cavity. Not a single case of HPV 16 positive has been reported in adolescents and HPV18 positive in children. Additionally, HPV 54 in children was the most frequently detected genotype and accounted for 2.5% of all subjects in this age group (3/118). However, when considering only positive cases in this age group, HPV 54 accounts for 33.3% (3/9). Data on the prevalence of the different HPV genotypes by age group are presented in Figure 2.

## 4. Discussion

The incidence of HPV varies in different populations and depends on the type of clinical material and the research method used [12]. The prevalence of asymptomatic HPV in the oral cavity is still an important subject in the field of head and neck carcinogenesis. Oral HPV infections are the result of sexual behavior; however, recent evidence suggests a possible mouth-to-mouth transmission through saliva [3]. The presence of HPV genotypes in the oral cavity in various studies has been estimated up to 20% [6,13,14,15,16,17,18]. In our study, the incidence of HPV was low and amounted to 11.5% (53/461). Additionally, the highest prevalence of HPV in the oral cavity was noted in the oldest subjects. In the 20–39 and 40–59 age groups, the presence of oral HPV may be caused by sexual activity. Similar to our data, studies in other countries have found that the age group around or over the age of 60 has the highest HPV presence in the oral cavity [6,19,20]. For adults over the age of 60, the number of positive oral HPV cases may be associated not only with sexual habits, but also with decreased immunity. Our study found the lowest presence of HPV in children (7.6%). Syrjänen [3] shows a wide variation (4–87%) in the estimation of the presence of oral HPV in this age group. HPV mucosal infections, unlike HPV cutaneous infections, are considered sexually transmitted. However, HPV has also been found in infants and children in the oral mucosa and genitalia suggesting non-sexual transmission. The perinatal route is considered to be the most likely cause of HPV in newborns. In addition, it was shown that children from HPV-positive mothers were 33% more susceptible to HPV infection than children born by HPV-negative mothers, and the risk was even higher when only high-risk genotypes were considered [3]. Moreover, as Syrjänen [3] points out, HPV can be acquired at an early age and remain dormant for years without developing any clinical changes. In our study, as in many others, the presence of HPV in the oral cavity was higher in males than in females [20,21,22,23,24]. This may be justified by the fact that biological and behavioral differences may increase the sensitivity of men to HPV infection [6].

HPV is associated with many different cancers. One of them is oropharyngeal cancer, which is one of the most common subtypes of head and neck cancers, with significant morbidity and mortality. Recently, HPV has been recognized as an etiological factor in this neoplasm [9]. The incidence of this cancer is increasing, so the information on the presence of individual HPV genotypes in healthy people is prognostically and diagnostically significant. Head and neck tumors associated with high-risk HPV have been shown to occur in younger patients who were not exposed to risk factors, including alcohol and tobacco use, and are associated with a better prognosis [11]. Tobacco and alcohol have genotoxic, pro-inflammatory and immunosuppressive effects that may increase the risk of HPV infection and its persistence, especially in the oral cavity. In Poland, oral and oropharyngeal cancer accounts for 3.8% of male and 1.3% of female cancers. About 90% of all cancers located in the oral cavity are squamous cell carcinomas, and their etiology is multifactorial. It is estimated that 10–30% of head and neck cancers are related to HPV infection, and the literature on the role of oncogenic HPV genotypes, especially HPV 16 and HPV 18, in these diseases is abundant [12,25,26,27]. Of the highly oncogenic types involved in head and neck carcinogenesis, HPV 16 is by far the most common, with over 80% occurring in oropharyngeal cancers followed by HPV 18 (3%) [28,29]. Overall, in our study, the prevalence of HPV 16 in the oral cavity was almost 0.9% (4/461). This data reported that adults had the highest presence of both HPV 16 (0.65%) and HPV 18 (0.4%) in the oral cavity. There has been a single case of HPV 16 in children and one case of HPV 18 in youth. Kreimer et al. [29], in their work, found that asymptomatic oral HPV 16 infection occurred in 1.3% of 3977 subjects. As Hearnden et al. [10] points out, many studies have limited the analysis to HPV 16 and HPV 18 infections, taking into account their predominance in oropharyngeal cancer cases. However, in our study, as in the study by Hearnden et al. [10], the number of cases with type 16 or 18 was low. Moreover, our study reported an increased presence of another high-risk type, HPV 45, especially in adults. Shigeishi et al. [30] in a study of 22,756 patients showed that the presence of oral HPV was 5.5%. In addition, the same study reported the presence of oral HPV at 2.2% for low-risk HPV, 2.7% for high-risk HPV, and 1.0% for HPV 16. The presence of low-risk oral HPV (HPV 6, HPV 11, HPV 54) in our study was 5.4% (25/461). The low-risk HPV types are associated with benign lesions, such as warts, precancerous lesions, and laryngeal papillomatosis [1].

Cancer prevention is important, as confirmed by recent data suggesting that oropharyngeal cancer incidence is increasing and may be due to the presence of oncogenic HPV types. In addition, the effectiveness of prophylactic HPV vaccination is less known for oropharyngeal cancer than for anogenital or cervical cancer [9]. Therefore, it is significant to assess the presence of HPV genotypes in the oral cavity among a healthy population. 

In conclusion, our study showed that the presence of oral HPV (both oncogenic and non-oncogenic) was low in the healthy population living in south-eastern Poland. The number of positive HPV cases was higher with the age of the subject. Moreover, HPV was more frequently detected in males and in people living in urban areas. It should be emphasized that further socio-epidemiological studies are needed to broaden the knowledge about the presence of oral HPV in the oral cavity. Our study has several limitations, such as the lack of information on risk factors that could be associated with the presence of specific genotypes in the studied population, such as smoking, alcohol consumption, sexual behavior, vaccination status, or piercing in the oral cavity. It should be noted that the detection of HPV genetic material depends on the method of collecting the material from the oral cavity, such as saliva or swab. In addition, various PCR methods are used to detect HPV DNA. It should be noted that in most studies on the presence of HPV, samples are taken from visible lesions in the oral cavity, while, in our study, the material was obtained from a healthy oral cavity [17]. Moreover, the quality of clinical specimens may vary from trial to trial, which may affect the trial results. In our study, brush swabs from patients were used and then tested using the commercially available INNO-LiPA kit, which exhibits high sensitivity and specificity.

## 5. Conclusions

The presence of HPV in the oral cavity of healthy subjects in south-eastern Poland was low at 11.5%. Prevalence of HPV in the oral cavity was more often detected in the elderly, and the lowest HPV detection was in children. In addition, in our study, oral HPV was found more often in males than in females. The presence of HPV in the oral cavity was higher in people living in urban areas than in rural areas. Overall, the presence of high-risk HPV, such as HPV 16 and HPV 18, was low in our study. Moreover, the presence of low-risk HPV in the oral cavity of the study population was 5.4%.

Summarizing, this study provides information on the presence of HPV genotypes in the oral cavity of a healthy population in south-eastern Poland and investigates the relationship between socio-epidemiological features and the presence of HPV in the oral cavity in healthy subjects. HPV is the cause of many cancers and a better understanding of its epidemiology and infection-related characteristics is crucial for effective and targeted preventive measures. Further research is needed to investigate the presence of HPV in this part of Europe, and thus detect early and reduce oral lesions, HPV-related cancers, and other comorbidities.

## Figures and Tables

**Table 1:** Prevalence of HPV in oral cavity and socio-demographic features.

		HPV (+)		HPV (−)		Total	*p*
		*n*	%	*n*	%	*n*	
**Adults**		**34**	**13.8**	**212**	**86.2**	**246**	
Age	20–39	6	17.7	76	35.9	82	0.0062 *
	40–59	8	23.5	70	33.0	78	
	60+	20	58.8	66	31.1	86	
Sex	F	6	17.7	116	54.7	122	6 × 10^−5^ *
	M	28	82.3	96	45.3	124	
Place of residence	Urban	30	88.2	97	45.7	127	4 × 10^−6^ *
	Rural	4	11.8	115	54.3	119	
**Youth**		**10**	**10.3**	**87**	**89.7**	**97**	
Age	11–19	10	100.0	87	100.0	97	
Sex	F	4	40.0	41	47.1	45	0.6686
	M	6	60.0	46	52.9	52	
Place of residence	Urban	8	80.0	41	47.1	49	0.0489 *
	Rural	2	20.0	46	52.9	48	
**Children**		**9**	**7.6**	**109**	**92.4**	**118**	
Age	5–10	9	100.0	109	100.0	118	
Sex	F	1	11.1	60	55.1	61	0.0112 *
	M	8	88.9	49	85.9	57	
Place of residence	Urban	9	100.0	53	48.6	62	0.0030 *
	Rural	0	0	56	51.4	56	

* Statistically significant.

## Data Availability

The data presented in this study are available in the article.
